# High-Stakes Hormone: Vasopressin Use as a Last-Line Therapy for Shock in Pediatrics—A Narrative Review

**DOI:** 10.3390/reports8030117

**Published:** 2025-07-21

**Authors:** Marcin Sota, Daria Bramnik, Olivia Gudziewski, Ithamar Cheyne, Małgorzata Mikaszewska-Sokolewicz

**Affiliations:** 1 Anesthesiology and Intensive Care Scientific Circle English Division (ANKONAED), Medical University of Warsaw, 02-091 Warsaw, Poland; 2 Department of Anesthesiology and Intensive Care, Children’s Memorial Health Institute, 04-730 Warsaw, Poland

**Keywords:** vasopressins, shock, critical care, pediatrics, catecholamines, hemodynamics, terlipressin, treatment outcome

## Abstract

Background and Clinical Significance: Shock in pediatric patients remains a leading cause of morbidity and mortality, with refractory cases posing significant challenges. While catecholamines like norepinephrine and epinephrine are standard vasopressors, vasopressin (AVP) has emerged as a potential adjunct therapy. However, its role in pediatric shock remains controversial due to concerns about efficacy, safety, and appropriate use. This review assesses the current evidence on AVP in pediatric shock. Methods and Results: A comprehensive literature search was conducted using PubMed, Scopus, Web of Science, and Google Scholar, focusing on studies published in the last five years to capture recent advancements. Articles on AVP’s mechanism of action, pharmacokinetics, clinical applications, and safety were included. For background information, studies were not limited by publication date. AVP increases mean arterial pressure (MAP) and systemic vascular resistance (SVR) yet does not significantly reduce mortality. While AVP may be useful in catecholamine-resistant vasoplegia, its advantage over conventional vasopressors remains uncertain. Concerns about ischemic complications, myocardial dysfunction, and thrombocytopenia further limit its routine use. Conclusions: AVP may serve as an adjunct therapy in catecholamine-resistant vasoplegia, but safety concerns and unclear benefits restrict its routine use. Further research is needed to determine the optimal dosing, patient selection, and long-term outcomes. Until then, AVP should remain a last-line therapy when conventional vasopressors fail.

## 1. Introduction and Clinical Significance

Shock is characterized by inadequate tissue perfusion and oxygen delivery, leading to cellular dysfunction and organ failure. In pediatric patients, shock has been reported to arise from various etiologies, including septic, cardiogenic, hypovolemic, and distributive causes. Despite the growth in therapies for critical care in pediatrics, shock continues to have significant levels of morbidity and mortality. The traditional global mortality rate for pediatric septic shock was estimated to range from 20% to 40%, varying by resource availability and healthcare quality [1]. But the recent 2024 Phoenix sepsis criteria show lower mortality rates of 10–20% [2]. The primary mechanism of death in shock in pediatric patients is multi-organ dysfunction syndrome (MODS) [3]. Early and effective intervention is said to diminish the progression of these events. Standard therapies for shock include fluid resuscitation, inotropes, and vasopressors, but refractory cases often call for innovative approaches. AVP has been considered an option in such high-stakes scenarios. As a molecule with vasoconstrictive and antidiuretic properties, it increases SVR, retains fluid, and helps perfuse vital organs such as the brain, heart, and kidneys. These effects may help restore perfusion to vital organs in catecholamine-resistant shock, although this benefit must be balanced against the risk of ischemia in less critical vascular beds. The following review will explore the role of using AVP in pediatric population shock, addressing its mechanism of action, applications, and limitations.

## 2. Literature Search Strategy

A comprehensive literature search was conducted to evaluate the role of AVP in pediatric refractory shock, focusing on its mechanism of action, clinical applications, efficacy, safety, and limitations. The search utilized PubMed, Scopus, Web of Science, and Google Scholar, covering articles published through 2024. We primarily focused on articles from the last five years to capture the recent clinical advancements and emerging evidence on vasopressin use in pediatric shock. However, for general background information regarding its mechanism of action and historical context, the search was not restricted by publication date. The key search terms included “vasopressin”, “arginine vasopressin”, “terlipressin”, “pediatric shock”, “vasodilatory shock”, “catecholamine-resistant shock”, and “vasopressor therapy”. The selection criteria included systematic reviews, meta-analyses, randomized controlled trials (RCTs), retrospective cohort studies, case series, and relevant guideline statements. Articles that focused solely on adult populations without pediatric relevance or lacked clinical applicability were excluded.

## 3. Current Protocols for Pediatric Septic Shock Management

The early recognition of shock is essential to reducing morbidity and mortality, as emphasized by current guidelines. According to the 2020 Surviving Sepsis Campaign (SSC) [4], fluid resuscitation with balanced crystalloids is the first line. These fluids are associated with a lower risk of hyperchloremic acidosis, acute kidney injury, and overall mortality than crystalloids with higher chloride concentrations. To ensure appropriate fluid resuscitation, the guidelines recommend hemodynamic monitoring based on dynamic parameters for fluid responsiveness, while static parameters such as heart rate, blood pressure, and pulmonary capillary wedge pressure (PCWP) are considered less reliable [5]. If fluid resuscitation is insufficient, vasoactive medications are introduced. Epinephrine or norepinephrine are typical first-line choices, although the quality of evidence supporting their use remains low. The recommended initial dose for both agents is 0.1 mcg/kg/min, increasing to 1 mcg/kg/min based on the patient’s response. In cases where hypotension persists despite high doses of catecholamines and where the patient exhibits very high cardiac output (vasoplegia), AVP is recommended as an adjunct therapy for refractory shock [4]. As a potent vasoconstrictor, AVP helps improve blood pressure in vasodilated shock [6], counteracting pathological vasodilation and preserving organ perfusion [7].

The updated 2021 SSC guidelines for adults recommend adding AVP when MAP remains inadequate despite norepinephrine and suggest adding it before further increasing norepinephrine to high doses, to limit the catecholamine-related side effects [8]. For the pediatric population, the 2020 SSC guidelines [4] provide only a weak recommendation to add AVP or further titrate catecholamines in such cases.

Refractory septic shock in children is defined by high lactate levels, high vaso-inotrope doses, and associated myocardial dysfunction [5]. The SSC guidelines [4] stress the importance of closely monitoring lactate levels, urine output, and the clinical signs of perfusion to evaluate treatment effectiveness.

## 4. Mechanism of Action of Vasopressin

**Figure 1:**
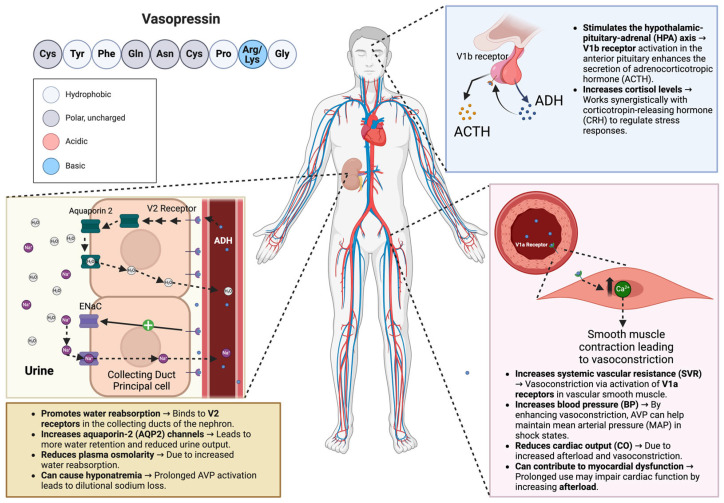
Mechanism of action of vasopressin. Description: Figure 1 illustrates the physiological effects of AVP in the body, highlighting its interaction with V1a, V1b, and V2 receptors and its role in vasoconstriction, water retention, and hormone regulation. ACTH—Adrenocorticotropic Hormone; HPA—Hypothalamic–Pituitary–Adrenal; ADH—Antidiuretic Hormone; SVR—Systemic Vascular Resistance; BP—Blood Pressure; MAP—Mean Arterial Pressure; CO—Cardiac Output.

AVP is a nine amino acid peptide hormone otherwise known as antidiuretic hormone (ADH). It is synthesized by magnocellular neurosecretory neurons in the hypothalamus as a pre-pro-hormone and later secreted by the posterior pituitary gland. AVP has been shown to act on three different receptor subtypes, V1aR, V1bR, and V2R, located throughout the body but most noticeably in the vascular smooth muscles, anterior pituitary, and renal collecting ducts, respectively (Figure 1). By binding to the V1aR receptor on vascular smooth muscles, AVP causes the activation of the phospholipase C signaling pathway, increasing systemic vascular resistance and blood pressure. The V1bR receptor is key in regulating the hypothalamic–pituitary–adrenal (HPA) axis, and AVP is found predominantly in the anterior pituitary. When AVP binds with V1bR, it stimulates the release of adrenocorticotropic hormones. The V1bR receptor forms a synergistic relationship with the corticotropin-releasing hormone, which controls ACTH secretion, affecting cortisol levels and keeping the body in balance under normal conditions or pressure [9,10]. When AVP binds to the V2R receptors expressed in the nephron, it starts a cascade that leads to the phosphorylation of aquaporin-2 channels and insertion into the apical membrane of collecting duct cells. This action results in increased water reabsorption [11]. Physiologically, this mechanism’s primary function is controlling plasma osmolarity, and its influence on vasomotor tone is of minor importance in healthy individuals. However, studies have shown that AVP plasma levels are inappropriately low in vasodilatory shock and that AVP deficiency contributes to vasodilatory septic shock hypotension [12]. Moreover, other studies have found that after the initial increase in AVP’s plasma levels in severe septic infection, it decreases along with post-pituitary storage [13,14].

## 5. Pharmacologic Comparison of Vasopressin and Catecholamines

**Figure 2:**
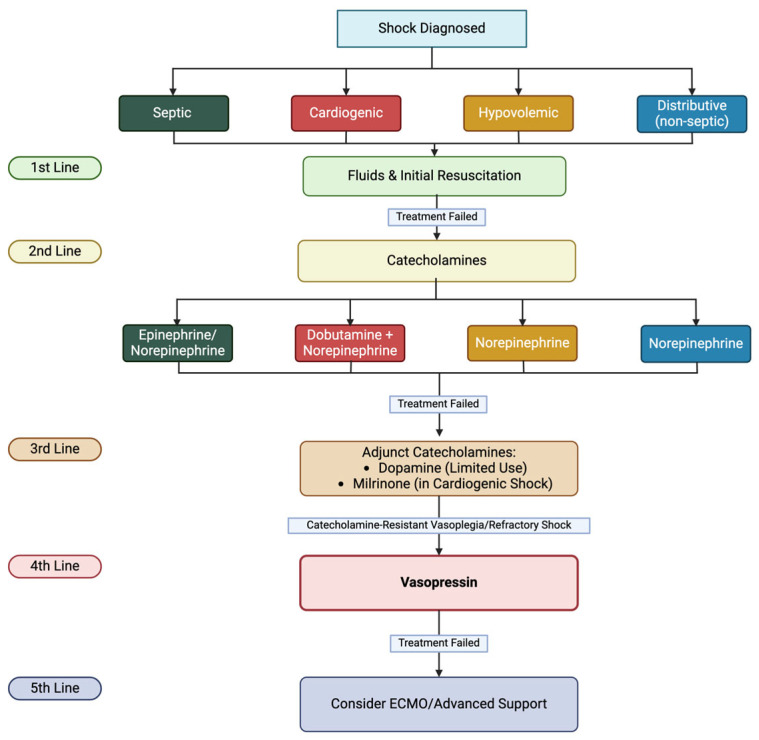
The use of AVP in shock management in pediatric patients. Description: Figure 2 presents a structured flowchart outlining the stepwise treatment of different types of shock, including septic, cardiogenic, hypovolemic, and distributive shock, from initial resuscitation to advanced interventions. Glossary: ECMO—Extracorporeal Membrane Oxygenation.

Currently, norepinephrine or epinephrine are used as a first-line agent in the management of septic shock [4]. A potent alpha-1 agonist produces significant vasoconstriction and stimulates beta-1 receptors, enhancing cardiac output [15]. Epinephrine is the first-line treatment for anaphylactic shock and targets all adrenergic receptors [16]. Due to its dose-dependent activation mechanism, epinephrine predominantly stimulates alpha receptors in high doses. In addition, it stimulates beta-receptors, leading to marked increased cardiac output. The third catecholamine used in shock, dobutamine, is the first line of treatment in cardiogenic shock due to low cardiac output [17]. Also, it is used as an adjunct treatment in combination with norepinephrine in cases of mixed shock. By stimulating the beta-1 receptors, dobutamine exhibits strong positive inotropic and chronotropic effects and a mild beta-2 response that causes dissociation. Therefore, dobutamine can increase cardiac output without causing significant vasoconstriction, which is particularly useful in cardiogenic and cardiogenic shock [18]. Dopamine has been used in cardiogenic shock and bradycardia shock. However, its use declined due to a higher risk of arrhythmias compared with norepinephrine [19]. Unlike catecholamines, AVP acts via the V1a, V1b, and V2 receptors and does not stimulate beta-1-adrenergic receptors. Catecholamines activate beta-1 receptors, increasing heart rate, contractility, and myocardial oxygen demand, which can cause a predisposition to arrhythmias. By reducing catecholamine requirements, vasopressin may thus help minimize these adverse cardiac effects [20]. Additionally, AVP is particularly of value when desensitization or downregulation occurs in cases of prolonged catecholamine use in shock [17]. Animal studies have shown that AVP can maintain mean arterial pressure in states where catecholamine resistance has occurred or where AVP could be potentially used as a last-line treatment [21,22,23,24]. Figure 2 shows the rationale of vasopressors use in shock treatment. 

## 6. Comparison Between Arginine Vasopressin and Terlipressin

AVP and TP are different preparations of the vasopressin hormone used in managing pediatric refractory shock. Understanding their pharmacological differences is crucial for optimizing treatment strategies. Table 1 [25] compares AVP and TP across various parameters relevant to their use in pediatric refractory shock.

While AVP and TP are utilized in pediatric refractory shock, they differ in receptor selectivity, half-life, administration routes, and dosing regimens. TP’s longer half-life allows for intermittent dosing, whereas AVP requires continuous infusion due to its shorter duration of action. These distinctions are essential when tailoring therapy to individual patient needs.

## 7. Limitations of Vasopressin Use

**Figure 3:**
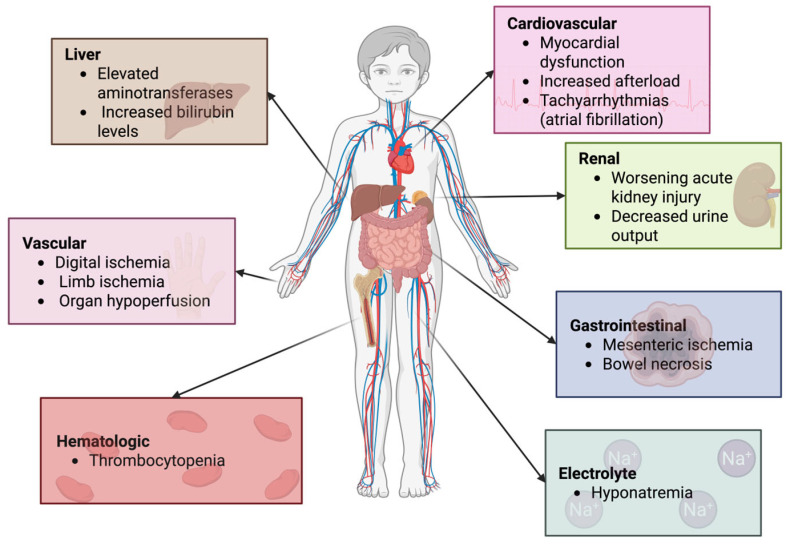
Adverse effects of vasopressin. Description: Figure 3 maps the systemic adverse effects of vasopressin across various organ systems, including cardiovascular, renal, vascular, hematologic, hepatic, and gastrointestinal complications.

Although AVP has many benefits in critical care settings, we must not discount the limitations when choosing AVP and its analogs in life-saving situations [26]. Due to its strong vasoconstrictive properties, AVP can lead to adverse effects such as increased systemic vascular resistance and afterload, reduced oxygen delivery, impaired tissue perfusion, and ischemic tissue injury (Figure 3). Reported complications include thrombocytopenia, elevated aminotransferase activity, and increased bilirubin levels (Figure 3). These effects appear to be dose-dependent, occurring more frequently at AVP doses exceeding 10 mU/kg/min or TP doses above 2 µg/kg/h. However, conflicting data exist regarding these risks, making it unclear whether the hemodynamic changes are an adaptive response to stabilized blood pressure or if impaired tissue perfusion is a consequence of disease severity rather than a direct adverse effect of AVP or concurrent catecholamine administration. Systematic reviews of adult and pediatric trials have thus far found no significant differences in the adverse event rates between AVP, TP, and other catecholamine-based vasoactive agents [27,28]. The administration of AVP and catecholamines (e.g., norepinephrine) typically involves continuous intravenous infusion, requiring advanced hemodynamic monitoring to titrate doses effectively [29].

Cost and availability can pose challenges in resource-limited settings, where vasopressors like norepinephrine may be more accessible and affordable compared with AVP. Therefore, there have been multiple initiatives to re-assess AVP formulation. For example, Kelly et al. [30] explored cost saving methods by reformulating standard AVP infusions to reduce waste. The new standard formulation adopted a concentration of 20 units/100 mL. As a result, this indicated a 38.7% decrease in AVP utilization, resulting in a cost decrease of 77,214.23 USD (based on 2019 cost estimates). In addition, a year later, Sizemore et al. [31] assessed a one-month period before and after changing the formulation to 20 units/100 mL, where each 20-unit vial of AVP cost 183.21 USD at the time. As a result, there was a saving of 366.42 USD per patient (175,881.60 USD annually). In conclusion, changing the AVP formulation proved to be a significant cost saving method.

## 8. Clinical Evidence

A meta-analysis by Masarwa et al. evaluating AVP and TP for refractory shock in the pediatric population revealed no significant impact on mortality or length of stay in the pediatric intensive care unit (PICU) compared with conventional therapy. While these therapies improved the hemodynamic parameters, including MAP and heart rate, these benefits did not translate into better survival outcomes. These findings are consistent with AVP’s known vasoconstrictive effects and risk of ischemia. The trial sequential analysis suggested that more extensive studies would be needed to confirm the findings, as the current evidence remains inconclusive [32]. The implications of this study indicate that while AVP/TP may offer short-term hemodynamic stabilization, their role in improving clinically meaningful outcomes in shock remains uncertain. The high rates of mortality and adverse events seen in the studies reflect the severity of refractory shock, underscoring the need for further research to refine patient selection and optimize treatment strategies [32].

The meta-analysis by Farias et al. discussing the effects of AVP infusion after pediatric cardiac surgery included a systematic review to identify studies concerning this topic. Additionally, six studies involving 160 patients were included for endpoints during the first 2 h of infusion, where AVP, infused at a dose of 0.75 mU/kg/min, was found to cause increased mean, systolic, and diastolic blood pressures, as well as lower heart rates at the 2 h mark [33]. Moreover, eight studies involving 338 patients were included to examine the effects at 24 h. AVP infusion, at the dose of 0.57 mU/kg/min, had a lower inotrope score and decreased central venous pressure. In this section, three studies compared data before and after the infusion. The remaining five patients were compared in two groups, one receiving the infusion and the other not. Those studies found that heart rate and blood pressure initially changed, whereas inotrope score, central venous pressure, fluid balance, and lactate levels changed later [33]. The findings were varied in the subset analyses of children with functionally univentricular hearts and neonates. No significant associations were present in the first two hours of the infusion. However, after 24 h, children with functionally univentricular hearts experienced decreased central venous pressures and reduced serum lactate concentrations. In neonates, there was a decrease in inotrope score and an increased fluid deficit [33]. The findings were found to be significant as pediatric patients are at risk for low cardiac output syndrome after cardiac surgery. In addition, studies have indicated an AVP deficiency in children after cardiac surgery. Compared with other common treatments, like dopamine, dobutamine, epinephrine, norepinephrine, or milrinone, AVP is especially appealing in vasodilation as it does not directly impact inotropy and myocardial oxygen consumption. Finally, AVP may help reduce the degree of capillary leak in the postoperative setting, reducing the need for fluid resuscitation postoperatively. The study has limitations, including a lack of patient-level data and heterogeneity in the pooled analyses. Despite this, those findings can guide future research [33].

Choong et al. conducted a study assessing the effectiveness and safety of using low-dose AVP as an adjunct therapy in pediatric vasodilatory shock [34]. In this double-blind, placebo-controlled trial conducted at seven pediatric critical care units in Canada, 65 children with vasodilatory shock were randomly enrolled into two groups, one that received AVP (0.5–2 mU/kg/min) and the other that received a placebo, in addition to standard vasoactive agents. The primary outcome, time to vasoactive-free hemodynamic stability, was not different between the treatment groups (AVP: 49.7 h; placebo: 47.1 h, *p* = 0.85). The secondary outcomes, such as mortality, organ-failure-free days, ventilator-free days, and length of stay, also did not demonstrate significant advantages. Mortality was higher in the AVP group (30% vs. 15.6% among the placebo group, *p* = 0.24), but the findings were not statistically significant. This trial showed no clear clinical benefit of AVP in pediatric vasodilatory shock. The doses investigated could not improve end organ perfusion indices like serum lactate concentration or urine output despite an increased MAP following the administration of AVP. The numbers of adverse events and serious adverse events were similar between the two groups. The authors proposed that the reason for failed treatment with AVP is the low impact of the AVP due to a decrease in AVP receptor responsiveness. The authors reason this by observing experimental models of septic shock, the suboptimal timing of administration, or the transition of certain patients from warm to cold shock due to a lack of real-time invasive hemodynamic or echocardiographic measurements. They have suggested that since no benefit was shown and since AVP could be harmful, the routine use of AVP in pediatric vasodilatory shock cannot be recommended based on the findings of this study [34].

## 9. Limitations

As a narrative review, this article is inherently subject to certain methodological limitations. While we aimed to provide a broad and up-to-date synthesis of the evidence regarding vasopressin use in pediatric shock, the absence of a structured systematic review protocol introduces the possibility of selection bias. The articles were chosen based on relevance and recent publication rather than formal inclusion and exclusion criteria, which may have influenced the comprehensiveness of the included data. Additionally, we did not perform a formal risk of bias assessment for the studies included, as this is outside the typical scope of narrative reviews. The variability in study designs, populations (e.g., neonates, postoperative patients), and outcome definitions further complicates comparisons and limits generalizability. Lastly, many of the studies cited were observational or small-scale trials, limiting the strength of the evidence base. These constraints highlight the need for future well-designed, large-scale randomized controlled trials to better evaluate vasopressin’s role in pediatric shock management.

## 10. Conclusions

The use of arginine vasopressin (AVP) in pediatric refractory shock remains a subject of ongoing investigation. While AVP has been shown to improve hemodynamic parameters such as mean arterial pressure (MAP) and systemic vascular resistance (SVR), current evidence does not support its ability to reduce mortality or improve long-term outcomes. Moreover, its use is associated with potential adverse effects, including digital and mesenteric ischemia, myocardial dysfunction, hyponatremia, and thrombocytopenia. These risks, along with the variability in study populations and dosing regimens, limit the routine implementation of AVP in pediatric critical care.

In clinical practice, AVP should not be considered a first-line vasopressor for pediatric shock. Instead, it may be considered as a last-line adjunct in catecholamine-resistant vasoplegia, particularly in cases with profound vasodilation and inadequate perfusion despite high-dose adrenergic support. Clinicians must weigh its short-term hemodynamic benefits against its adverse effect profile and ensure the close monitoring of tissue perfusion and electrolyte status.

Future research should prioritize large-scale, randomized controlled trials that stratify patients by age group, shock subtype (e.g., septic, post-cardiac surgery), and AVP responsiveness. Optimal dosing strategies, the timing of initiation, and the long-term safety of AVP—particularly in neonates and low cardiac output states—remain key areas of uncertainty. Comparative trials between AVP and terlipressin may further clarify whether selective receptor activity or extended half-life confers a clinical advantage. Until such evidence is available, the use of AVP should remain selective and individualized.

## Figures and Tables

**Table 1:** Comparative pharmacological and clinical profiles of AVP and TP in pediatric refractory shock.

Parameter	Arginine Vasopressin (AVP)	Terlipressin (TP)
Type	Endogenous nonapeptide hormone.	Synthetic vasopressin analog. Prodrug, dependent on hepatic and renal metabolism, to transform into the active lysine vasopressin form.
Half-Life	Approximately 10–20 min.	Approximately 6 h.
Route of Administration	Continuous intravenous infusion.	Typically administered as intermittent intravenous boluses.
Dosing for Shock	Initiated at 0.1 to 0.3 mU/kg/min, titrated based on clinical response.	Administered at 20 µg/kg every 4 h; dosing may vary based on patient condition and response.
Onset of Action	Rapid onset within minutes due to continuous infusion.	Slower onset compared with AVP, corresponding with its longer half-life.
Mechanism of Action	It causes vasoconstriction via V1 receptor stimulation, leading to increased systemic vascular resistance and blood pressure, and it promotes renal water reabsorption through V2 receptors.	Acts predominantly on V1 receptors to induce vasoconstriction, increasing systemic vascular resistance and blood pressure; minimal effect on V2 receptors.
Metabolism/Elimination	Metabolized by hepatic and renal pathways, rapid clearance contributes to a short half-life.	Metabolized to lysine vasopressin, slower metabolism results in prolonged duration of action.

## Data Availability

Not applicable.

## Abbreviations

The following abbreviations are used in this manuscript:

ACTH	Adrenocorticotropic Hormone
ADH	Antidiuretic Hormone
AVP	Arginine Vasopressin
CO	Cardiac Output
ECMO	Extracorporeal Membrane Oxygenation
HPA	Hypothalamic–Pituitary–Adrenal
MAP	Mean Arterial Pressure
MODS	Multiple Organ Dysfunction Syndrome
PCWP	Pulmonary Capillary Wedge Pressure
PICU	Pediatric Intensive Care Unit
RCT	Randomized Controlled Trial
SVR	Systemic Vascular Resistance
TP	Terlipressin
